# Supplementary material for: Reconciling Mining with the Conservation of Cave Biodiversity: A Quantitative Baseline to Help Establish Conservation Priorities
Source: PLoS One. 2016 Dec 20;11(12):e0168348. doi: 10.1371/journal.pone.0168348 (PMC5173368; doi:10.1371/journal.pone.0168348)
Supplement: S1 Dataset — (ZIP) [file pone.0168348.s002.zip › Taxa/Serra Norte/SN_2013/N4WS/Lista N4WS-39.pdf]

CAVIDADE N4WS-0039

Classe	Ordem	Fam/Outros	Gên/Outros	Espécie	S-U	U-S	Zon.
Arachnida	Acari	Mesostigmata		sp.1	X		E
Arachnida	Amblypygi	Phrynidae	*Heterophrynus*	*longicornis*	2		E
Arachnida	Araneae	Ctenidae		jovem		1	E
Arachnida	Araneae	Ochyroceratidae	*Speocera*	sp.1	X	X	E/P
Arachnida	Araneae	Oonopidae		sp.2		X	E
Arachnida	Araneae	Pholcidae	*Mesabolivar*	*eberhard*	X	X	E
Arachnida	Araneae	Scytodidae	*Scytodes*	jovem		1	P
Arachnida	Araneae	Theraphosidae		jovem		1	P
Arachnida	Araneae	Theridiidae		jovem	X		E
Arachnida	Opiliones	Cosmetidae	*Roquettea*	*singularis*	6		E
Arachnida	Opiliones	Sclerosomatidae	*Prionostema*	sp.1	X	X	E/P
Arachnida	Pseudoscorpiones	Chernetidae	*Spelaeochernes*	sp.1	X		E
Arachnida	Pseudoscorpiones	Chthoniidae	*Pseudochthonius*	sp.1	X	X	E/P
Arachnida	Pseudoscorpiones	Olpiidae		jovem	X		E
Arachnida	Schizomida	Hubbardiidae	aff. *Rowlandius*	sp.1		X	P
Chilopoda	Scolopendromorpha	Scolopocryptopidae	*Newportia*	sp.1	1		E
Diplopoda	Polydesmida	Pyrgodesmidae		sp.1	X		E
Entognatha	Collembola	Entomobryidae		sp.1	X		E
Entognatha	Collembola	Entomobryidae		sp.7		X	E
Entognatha	Collembola	Paronellidae		sp.1	X	X	E
Entognatha	Diplura	Campodeidae		sp.1	X	X	E
Insecta	Blattodea			jovem	2	1	E
Insecta	Coleoptera	Staphylinidae		sp.54	X		E
Insecta	Diptera	Psychodidae	*Sciopemyia*	*sordellii*	X	X	E/P
Insecta	Diptera	Sciaridae	*Bradysia*	sp.	X		E
Insecta	Diptera	Tipulidae	Tipulinae	sp.	X	X	E/P
Insecta	Hymenoptera	Formicidae	*Camponotus*	*atriceps*		1	P
Insecta	Hymenoptera	Formicidae	*Dolichoderus*	aff. *bispinosus*	X		E
Insecta	Hymenoptera	Formicidae	*Nylanderia*	sp.1	X		E
Insecta	Hymenoptera	Formicidae	*Nylanderia*	sp.3		X	E
Insecta	Hymenoptera	Formicidae	*Pachycondyla*	*striata*	2	1	E/P
Insecta	Hymenoptera	Formicidae	*Pheidole*	sp.2	X		E
Insecta	Hymenoptera	Formicidae	*Strumigenys*	sp.1	X		E
Insecta	Isoptera	Rhinotermitidae	*Heterotermes*	sp.	X		E
Insecta	Isoptera	Termitidae	*Embiratermes*	sp.		X	E/P
Insecta	Isoptera	Termitidae	*Nasutitermes*	sp.	X		E
Insecta	Lepidoptera	Noctuoidea		sp.1	2		E
Insecta	Lepidoptera	Noctuoidea		sp.2	X		E
Insecta	Lepidoptera	Noctuoidea		sp.4		X	P
Insecta	Neuroptera	Myrmeleontidae		jovem	1		E
Insecta	Orthoptera	Phalangopsidae	*Paraclodes*	sp.	27	8	E
Insecta	Psocoptera	Lepidopsocidae		jovem	X		E
Insecta	Psocoptera	Ptiloneuridae	*Ptiloneura*	sp.1	X	X	E
Insecta	Thysanoptera	Phlaeothripidae		sp.2	X		
Insecta	Thysanoptera	Thripidae		sp.1		X	P
Gastropoda	Pulmonata	Systrophiidae	*Happia*	sp.	X		E
Malacostraca	Isopoda	Armadillidae		sp.1	X	X	E
Amphibia	Anura	Strabomantidae	*Pristimantis*	cf. *fenestratus*	9		E
Mammalia	Chiroptera	Emballonuridae	*Peropteryx*	sp.	1		E

Mammalia	Chiroptera	Furipteridae	*Furipterus*	*horrens*	1		E
Mammalia	Chiroptera			sp.		1	E
